# *QuickStats:* Percentage of Births in Which Women Received Prenatal Care in the First Trimester of Pregnancy, by State — United States, 2024

**DOI:** 10.15585/mmwr.mm7525a3

**Published:** 2026-07-02

**Figure:**
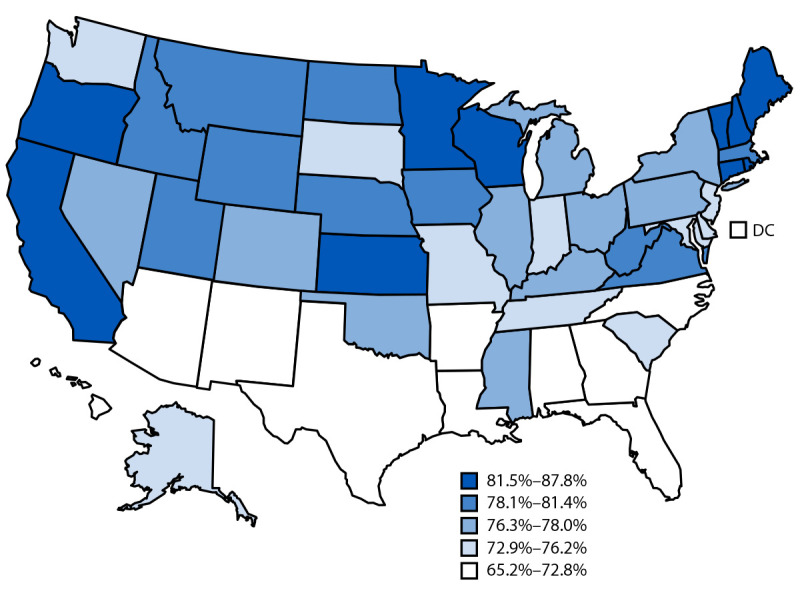
For births in the United States in 2024, a total of 75.5% were among women who received prenatal care beginning in the first trimester of the pregnancy. Rates ranged from 65.2% in Florida to 87.8% in Vermont.

**Supplementary Table:** https://stacks.cdc.gov/view/cdc/257025?preview=1#tabs-3

**Source**: National Center for Health Statistics, National Vital Statistics System, Natality Data 2024. NVSS - Birth Data

For more information on this topic, CDC recommends the following link: Pregnancy | Office on Women's Health.

